# Otology practice during COVID-19 era: a review of current practice

**DOI:** 10.1186/s43163-020-00055-9

**Published:** 2020-11-25

**Authors:** Moustafa M. Dawoud

**Affiliations:** 1 Otorhinolaryngology Department, Faculty of Medicine, Menoufia University, Shebin Elkom City, Menoufia, Egypt; 2 Otorhinolaryngology Department, Sheffield Teaching Hospitals, Sheffield, UK

**Keywords:** COVID-19, Otology, Aerosol-generating procedures

## Abstract

**Background:**

The novel coronavirus started as an outbreak in Wuhan, China, in December 2019.The outbreak was declared a pandemic by the WHO on 12 March 2020. The virus is called SARS-CoV-2, and the virus-induced disease is called COVID-19. The infection spreads via droplets or direct contact with contaminated surfaces. Airborne transmission occurs during aerosol-generating procedures on patients. Many otologic procedures are considered AGPs and therefore require precautions to protect staff and patients and minimize transmission of the disease.

**Main body:**

Outpatient otology activity has seen changes, including virtual clinics and limitation of face-to-face consultations, to ensure safety. Powered instrumentation should be avoided during surgical procedures unless necessary or replaced with other tools, and if performed, enhanced personal protective equipment (PPE) must be used. Ear, nose, and throat (ENT) examination is recommended for any patient with full PPE in place except for consultations done without examination. Systemic steroid administration for treating conditions such as Bell’s palsy and sudden sensorineural hearing loss should be discussed with both the patient and infectious diseases specialist to weigh risks against benefits. Triaging of patients and prioritization is unavoidable during the pandemic and even after due to the limitations of clinic and theater time. All emergency/urgent cases are considered potentially COVID-19 positive. For the semi-urgent and all elective cases, COVID-19 testing 48 h prior to surgery, strict quarantine awaiting test results, and repeat testing on day of surgery if rapid tests are available are the precautions suggested. Different measures should be in place to minimize staff potential exposure intraoperatively.

**Conclusions:**

Otology practice has been affected by the COVID-19 pandemic. Various measures are in place to ensure the delivery of safe and effective service for patients and health care workers.

## Background

The novel coronavirus started as an outbreak in Wuhan City located in Hubei Province, China, in December 2019 [1]. The outbreak rapidly progressed across the world and was declared a pandemic by the WHO on 12 March 2020. The virus is called SARS-CoV-2, and the virus-induced disease is called 2019 novel coronavirus disease (COVID-19) [2].

The mode of spread is via droplet infection or direct contact with a surface or an object contaminated with the virus. In fact, COVID-19 is not considered transmissible through the airborne route unless an aerosol-generating procedure (AGP) is performed on the patient [2].

## Main text

*Aerosol-generating procedures* have been identified by US Centers of Disease Control and Prevention (CDC) as procedures performed on patients who are more likely to produce higher rates of respiratory infectious aerosols than coughing, sneezing, talking, or breathing [3].

Health Protection Scotland also defined AGP as medical and patient care procedures that result in the creation of airborne particles (aerosols) that generate the potential for airborne transmission of infections that otherwise can only be transmitted by the droplet route [4].

Coughing and sneezing are not included in the list of AGPs. One calculation is that 99.9% of the volume of fluid is subject to gravitational influence in larger droplets, which moves just a short distance [5].

AGPs can be classified into respiratory and surgical procedures, bearing in mind that respiratory procedures aerosolize respiratory or upper airway secretions. These have a higher viral content and present a greater risk of transmission than surgical procedures that aerosolize blood and tissue fluid [6].

Procedures that carry the risk of aerosol generation as a source of respiratory pathogens include positive pressure ventilation (BiPAP and CPAP), endotracheal intubation, airway suction, high-frequency oscillatory ventilation, tracheostomy, chest physiotherapy, nebulizer treatment, sputum induction, and bronchoscopy [7].

The current recommendation by Public Health England is to have airborne precautions in risky areas where AGPs are performed on a regular basis if any suspected COVID-19 patients are present, including intensive care units, operating theaters while AGPs are performed, and emergency department resuscitation bays [2].

The levels of personal protective equipment (PPE) during AGP are variable among countries. In a scientific statement on 29 March 2020, the WHO recommended droplet and contact precautions routinely and airborne precautions for AGPs [8].

The accepted evidence-based recommendations suggest taking additional infection prevention steps for particular AGPs conducted on patients with suspected respiratory infection, conducting aerosol-generating procedures in a single room with a minimum number of staff present, and employing the most qualified staff to conduct AGP quickly and efficiently [7]. Airborne precautions consist of fit-tested and fit-checked high filtration mask, goggles or visor, long-sleeved fluid-repellent gown, and gloves. Increasingly, most guidance includes the use of FFP2 masks if FFP3 masks are not readily available [9, 10].

The World Health Organization (WHO) stressed on the fact that FFP2/3 and N95 masks should not be used unless they fit the face well and achieve a seal. All relevant staff members should undertake individual mask fit testing before they are worn on clinical duty [11]. It is recommended to provide a “buddy scheme” with an observer using a checklist to ensure that PPE is placed on (donning) and removed (doffing) properly. Training on donning and doffing of PPE before patient care is important for personnel and patient safety [6, 7]. Some authors recommended that further protection should be used during performing AGPs, including wearing coveralls inside the gown, water-resistant boot covers, and face shield outside the goggle, aiming to minimize skin exposure. Additionally, wearing double gloves is recommended to avoid tearing hazard, while the inner layer can provide greater security while putting off the external equipment. Invasive airway procedures such as tracheotomy are at the highest risk and require a powered air-purifying respirator (PAPR) to provide ultimate protection from aerosols [12].

Sputum and upper airway secretions harbor the largest viral load of SARS-CoV-2. While viremia can occur, blood-borne infection is not yet considered a major transmitting source [13]. High viral titers may occur in the oropharynx of infected patients at or before the onset of symptoms and may also occur in asymptomatic individuals [14]. The middle ear mucosae and even the mastoid in terms of histological epithelium can be regarded as part of the upper airway. In 1998, coronaviruses were detected in otitis media tissue and fluid samples [15]. Wanna and colleagues in Mount Sinai University Hospital, USA, reported sampling the mastoid and middle ear simultaneously with a nasopharyngeal swab in a 54-year-old lady who was diagnosed a month earlier with COVID-19-positive swab. Both sampling results came back negative [16]. This evidence is weak because of lack of sampling during active disease status, as well as being a single case report.

### COVID-19 and Otorhinolaryngology practice

Despite all precautionary measures followed, health care workers (HCW) represented a percentage of 3.8 to 20% of the infected population, with 14.8% of those suffered severe or critical disease [17, 18]. The first recorded physician to die in Wuhan, China, relating to COVID-19 was an otolaryngologist on 25 January 2020 [19].

Due to the wide range of examinations and operating procedures they conduct in the airways, ear, nose, and throat (ENT) specialists are especially at risk of contracting the disease [20], being in close contact with respiratory droplets or aerosols caused by patient’s involuntary coughing, sneezing, and deep breathing, while performing endoscopy for nose and larynx, controlling epistaxis, and other various procedures [20].

There is a wide spectrum of COVID-19-related ENT presentations, including completely asymptomatic individuals and mild upper respiratory tract symptoms such as sore throat, rhinorrhea, nasal obstruction, and tonsillitis [21]. The isolated sudden onset anosmia (ISOA) is currently recognized as the sole presentation of COVID-19 in certain patients, accompanied occasionally with dysgeusia [22]. As a result of the high risk related to ENT procedures, ENT UK, the official advisory body for ENT in the UK, recommended stopping all non-essential ENT examinations or procedures during the pandemic to minimize exposure of HCW to the disease, with concurrent protection of patients who are no longer in physical contact with the hospital environment unless necessarily needed [23].

Due to this approach, telephone consultations and virtual clinics have been implemented as a temporary approach to carry out consultations. In fact, this mode of consultation has proven efficacy in certain cases, such as follow-up patients whose clinical condition is stable or in case of long waiters. This has encouraged the ENT UK to recommend continuation of this type of consultation even after the pandemic is over [24]. Fieux and colleagues have also studied efficacy of the telephone consultation in ENT practice during the current pandemic environment in France, with regard to patient satisfaction. The study was questionnaire based and concluded that 87% satisfaction was achieved and stressed on the fact that lack of physical examination has not affected patients’ reflection on satisfaction [25]. This mode of consultation, although proved successful in developed countries, may not be applicable in developing countries which do not possess a well-established database of patients that facilitates pre-booking into clinics.

To date, there is no fast reliable testing scheme for the detection of a patient’s COVID-19 infection status. This has led to a worldwide concept to postpone all elective cases and consider all emergent/urgent cases as potentially high risk for COVID-19 [23, 26]. All ENT procedures carrying a risk of potential aerosol generation, such as nasolaryngoscopy, transnasal endoscopy, and high-speed handpieces or ultrasonic devices, increase the risk of infection and should be avoided or performed within restricted indications [26]. It is inevitable that due to the current guidance and restrictions on performing any elective examination and operations, patients will present late with more advanced and complicated stages. This will mandate more emergency interventions as this continues over time [27].

### Adjustments to otology practice during the COVID-19 pandemic

Up to June 2020, the general consensus was that in case, ENT examination is needed for a patient to proceed only with enhanced PPE. This includes otoscopy, binocular microscopy, ear wax clearance, or microsuction with specific advice to use unfenestrated suction to reduce the chance of aerosolization [26, 28].

This guidance has been relaxed in the last version of the ENT UK guide for graduated return of elective activity released on 15 July 2020, stating that in consultations where no AGPs are performed, FFP3 masks and enhanced PPEs are not required [24]. However, the use of a fluid-resistant mask and/or a visor is still recommended. Patients are still expected to wear a mask during consultation as well as physicians. This may create a problem for deaf patients attending the clinic who are unable to lip read. This can be partially overcome if the physician wears a clear visor while maintaining the safe distance from the patient or the use of translation software that can offer electronically printed text for the patient to read.

Standard otoscopy should be avoided whenever possible and can be replaced by either microscopy or the use of video otoscopy with a remote screen [24].

There is a debate regarding systemic steroid administration for certain otological presentations, such as Bell’s palsy and sudden sensorineural hearing loss (SSNHL). It is suggested to carry out a discussion with infectious diseases specialists to establish a protocol that bears in mind the patient’s general condition and comorbidities that may increase their vulnerability with the use of systemic steroids. Due to the limited current data, some authors tend towards avoidance of steroid treatment [26, 28]. This concept has been questioned following the recent breakthrough that proved the efficacy of dexamethasone in the treatment of moderate and severe cases of COVID-19 [24]. The limited systemic effect and absorption of locally injected steroids via the intratympanic route support its utilization as a substitute for systemic treatment in cases of SSNHL, with advice against asking the patient to spit and not swallow following the procedure to avoid unnecessary aerosolization [28]. Dry mopping of a wet ear (either due to otitis externa or a perforated tympanic membrane) can be a safer approach for external auditory canal (EAC) clearance than microsuction [24].

Triaging of patients and prioritization is unavoidable during the pandemic and even after due to the limitations of clinic and theater time. These limitations arise from the necessity to reduce the number of patients in each clinic to apply rules of social distancing and allow time for proper sanitization, and donning and doffing of PPE. This also applies to the availability of theater slots, spacing between patients to allow time for proper team communication, briefing, instrument and device checking, donning, and doffing and to allow time for theater team to rest [29, 30].

### Prioritizing surgical procedures

Priorities of otological procedures have been classified by Topsakal et al. [23] into the following categories shown in Table 1.

**Table 1:** Priority of otologic surgery during the COVID-19 pandemic

**Priority: urgent**	
Life-threatening ear disease or related complications	
**Priority: within 48 to 72 h**	
Acute mastoiditis not responding to maximal medical treatment	
Acute mastoiditis with subperiosteal abscess	
Cranial and intracranial complications of cholesteatoma	
Barotrauma with evident perilymph fistula and SNHL	
Trauma to facial nerve, pinna	
Vestibular schwannoma with brainstem compression event	
**Priority: within 4 weeks**	
Cochlear implantation for SNHL because of meningitis	
Otologic neoplasia; awaiting decision of multidisciplinary team (MDT)	
**Priority: within 12 weeks**	
May seem safe for	
Cholesteatoma, uncomplicated and stable	
Cochlear implantation for pre-lingual profound SNHL	
Implantable hearing aids	
Non-life-threatening lesions requiring neurotologic procedures	
Ossiculoplasty, stapedoplasty, meatoplasty, tympanoplasty	

Another useful prioritization was suggested by Saadi et al. [26], which included a category of some elective cases that can be deferred indefinitely until newer guidance is issued. This includes tympanoplasty for dry/stable perforation, stapes surgery, ossicular reconstruction, adult cochlear implantation (except for postmeningitic cases), and bone anchored hearing aids, as all these procedures fall under the category of quality of life-related hearing rehabilitation [24, 28].

### Peri-operative considerations

All emergency/urgent cases are considered potentially COVID-19 positive [23, 26]. For the semi-urgent and elective cases, COVID-19 testing 48 h prior to surgery, strict quarantine awaiting test results, and repeat testing on the day of surgery if rapid tests are available are the precautions suggested by Saadi and colleagues [26]. False sense of security should be avoided upon obtaining negative results due to the limited reliability of current testing methods. A useful advice is to include a limited dose-screening CT scan of the chest during temporal bone scanning, which can reveal pulmonary changes even in asymptomatic patients; such changes are more diagnostic than PCR results in these patients [31].

Wherever applicable, the procedure should be performed in a well-ventilated negative-pressure environment with the doors closed. Only necessary staff is allowed to be present. If these settings are not readily available, ensuring rapid in-room air turnover is more important than if it is at negative or positive pressure [6, 32].

A very detailed team briefing is essential for safe, quick, and effective procedures. This communication is crucial, as contact between team members will be limited with full PPE worn during the procedure [29, 30].

Anesthetic techniques that minimize coughing along with pre-oxygenation with 100% oxygen and rapid sequence induction are highly recommended to avoid manual ventilation that carries a higher risk of aerosol generation [6, 32].

Proper hypotension delivered by the anesthesiologist will help keep bleeding to the minimum and minimize the risk of blood and bony microspicules’ aerosolization [23].

The most experienced otologist should perform these procedures in the absence of trainees to reduce surgical time and avoid unnecessary exposure. The theater team should also limit the number of door movements and hazardous entries of non-essential staff in theater. Markings on the doors with “COVID-19 contaminated” are necessary precautions to warn them [28, 29].

With regard to mastoid surgery, such as cortical mastoidectomy, modified radical mastoidectomy (MRM), or combined approach tympanoplasty (CAT), it is documented that bony microspicules resulting from high-speed drilling can penetrate the cornea in animal models and act as a source for viral transmission [32]. Therefore, powered instrumentation should be avoided unless necessary or replaced with other tools, such as chisel with curettes to remove the mastoid cortex, and if performed, PAPR or properly sealed eye protection is recommended [23, 33].

There is no evidence regarding the hazard of exposure to electro-cautery smoke or trans-oral laser resection-generated smoke, but taking appropriate precautions in these settings is advisable [20].

The use of piezoelectric drill has been suggested as another alternative for conventional drills with limited evidence regarding aerosol generation [34].

The surgeon should not consider the microscope as a shield against aerosolization. All PPE should be worn adequately, including eye protection with the possibility of wearing PARP if readily available [26]. Eye protection has been a persistent issue, especially with microscope use. Soft visors, tightly fitted glasses, and foam-surrounded glasses have all proven successful with different surgeons [24]. A modified face shield has been recently developed by an Australian team to meet the needs of ENT surgeons and abolish the risk of aerosol exposure. They developed a cheap model which can be reproduced on commercial level worldwide. It is primarily made of laser cut polyethylene terephthalate glycol (PETG) sheeting. It is custom made to fit the head light and increase clarity of vision through the shield [35].

Moreover, a two-layered microscope draping technique was suggested by ENT consultants in the UK and published by ENT UK, which employs the second layer of draping as a tent over the head of the patient to prevent the aerosol generation effect [36]. In a similar way, the American Academy of Otolaryngology-Head and Neck surgery (AAO-HNS) recently published a custom strategy to mitigate the risk of aerosol generation during mastoidectomy. The research team compared 3 different strategies for draping including no barrier draping, draping with a technique called OtoTent1, and draping with the OtoTent2 technique. The OtoTent2 prototype draping produces a three-dimensional space around the microscope lens. It contains two re-enforced ports for surgeon’s arms and a third port to introduce the drill and suction into the tent. They have concluded that OtoTent2 draping proved to have the least risk of aerosol dispersion and also recommended using a second suction during drilling and having a proper pause before removing the drill and the barrier draping to mitigate the risk furthermore [37].

The use of endoscopy can be another alternative, with a drawback of a single-handed technique. Additionally, the use of exoscope was proposed, with a limitation of its unavailability in most hospitals [23, 28]. The exclusive use of endoscope in treatment of chronic suppurative otitis media (CSOM) including cholesteatoma has been proposed in a recent publication by Ayache and his colleagues, as it provides a panoramic view of the middle ear, alleviates the need for drilling, and allows wearing goggles and even face shields without interference with surgeon’s vision of the surgical field [38]. This trans-canal endoscopic approach seems to be an excellent approach in case of limited cholesteatoma that does not require mastoid drilling for access and limits the need for mastoid drilling to more extensive cases.

Regarding the training opportunities, a study conducted on Italian trainees in a major tertiary referral hospital concluded that cadaveric dissection lab activities have proven efficacy as substitute to live surgery in a more safe and controlled environment. Attendance of live webinars and online lectures allowed trainees to exchange experience and explore other institutes and ORL schools around the world [39]. These strategies can be a temporary substitute to overcome the delay in trainee development and reduced theater time during the pandemic time.

In a recent breakthrough, on 15 August 2020, the American Food and Drug Administration (FDA) have approved the SalivaDirect test for rapid detection of SARS-CoV-2 from salivary samples. It is promoted to be a fast and reliable test, which can be a game changer on all aspects [40]. For such testing technique to be considered a success, it needs to be readily available internationally at low cost with sufficient supplies to cover the worldwide needs. If such a test is widely approved, it will have a direct impact on peri-operative assessment of patient’s COVID-19 status and may lead eventually to resume elective activity on a wider scale.

## Conclusions

1. Otology practice has been massively affected by the COVID-19 pandemic, which in turn will have its impact on both the short and long term on training opportunities and quality of service provided.
2. Major adjustments have been implemented worldwide to guarantee delivery of a safe yet effective service both for patients and health care workers.
3. Outpatient activity has seen changes that will remain in place in the long term, including virtual clinics, limitation of face-to-face capacity, and strict sterilization and airflow exchange measures.
4. Prioritization of surgical procedures is a must due to limited capacity and extra precautions in place.
5. Peri-operative precautions include self-isolation of the patient for 2 weeks and repeated testing until 48 h prior to surgery. Full PPE should be worn by staff during AGPs to limit the risk of viral transmission.
6. Employing different surgical approaches and alternative tools to avoid high-speed drilling is highly recommended, specifically in suspected or confirmed COVID-19 cases to avoid aerosol generation.

## Data Availability

Not applicable

## Abbreviations

AGP: Aerosol-generating procedure
BiPAP: Bi-level positive airway pressure
CDC: Center of Disease Control
COVID-19: Novel coronavirus disease
CPAP: Continuous positive airway pressure
CSOM: Chronic suppurative otitis media
CT: Computed tomography
EAC: External auditory canal
ENT: Ear, nose, and throat
FDA: The American Food and Drug Administration
FFP2: Filtering face piece 2 mask
FFP3: Filtering face piece 3 mask
HCW: Health care workers
MDT: Multidisciplinary team
N95: Face mask produced by 3M company
PARP: Powered air-purifying respirator
PCR: Polymerase chain reaction
PETG: Polyethylene terephthalate glycol
PPE: Personal protective equipment
SNHL: Sensorineural hearing loss
SSNHL: Sudden sensorineural hearing loss
WHO: World Health Organization
